# En-bloc Transplantation: an Eligible Technique for Unilateral Dual Kidney Transplantation

**Published:** 2012-08-01

**Authors:** M. Salehipour, A. Bahador, S. Nikeghbalian, K. Kazemi, A. R. Shamsaeifar, S. Ghaffaripour, M. A. Sahmeddini, H. Salahi, A. Bahreini, P. Janghorban, S. Gholami, S. A. Malek-Hosseini

**Affiliations:** *Shiraz Organ Transplant Unit, Nemazee Hospital, Shiraz University of Medical Sciences, Shiraz, Iran*

**Keywords:** Kidney transplantation, En-bloc transplantation, Dual kidney transplantation, Expanded criteria donor

## Abstract

Background: Kidney transplantation is the best available treatment for patients with end-stage renal disease.

Objective: To evaluate the en bloc anastomosis technique for unilateral dual kidney transplantation (DKT).

Methods: From May to October 2011, 5 patients (4 women and 1 man) with mean age of 31.8 years underwent unilateral DKT with this technique in which distal end of the aorta and proximal end of inferior vena cava (IVC) were closed with running sutures. Then, proximal end of the aorta and distal end of the IVC were anastomosed to internal (or external) iliac artery and external iliac vein, respectively.

Results: Post-operative course was uneventful. No vascular and urologic complications developed; all patient had acceptable serum creatinine at discharge time and up of 2–6 months of post-operation follow up.

Conclusion: Unilateral DKT is a safe method for performing DKT. The proposed en bloc anastomosis can improve the outcome of the graft by reducing the cold ischemia and the operation time.

## INTRODUCTION

Kidney transplantation is the treatment of choice for patients with end-stage renal disease (ESRD). The progress in surgical transplantation techniques and immunosuppression has prolonged patient and graft survival, but organ availability is still a major limitation for expanding renal transplantation programs. The disparity between the number of organs available for transplantation and the number of patients awaiting transplantation has led most transplantation centers to use organs from expanded criteria donors (ECD). Dual kidney transplantation (DKT) was developed to increase the use of kidneys from ECD.

Herein, we report on our initial experience with en bloc surgical technique of unilateral DKT.

## PATIENTS AND METHODS

From May to October 2011, five patients (four women and one man) with mean age of 31.8 (range: 11–48) years underwent DKT from deceased donor. The mean donor age was 15 (range: 2–61) years.

 Surgical technique 

**Figure1:**
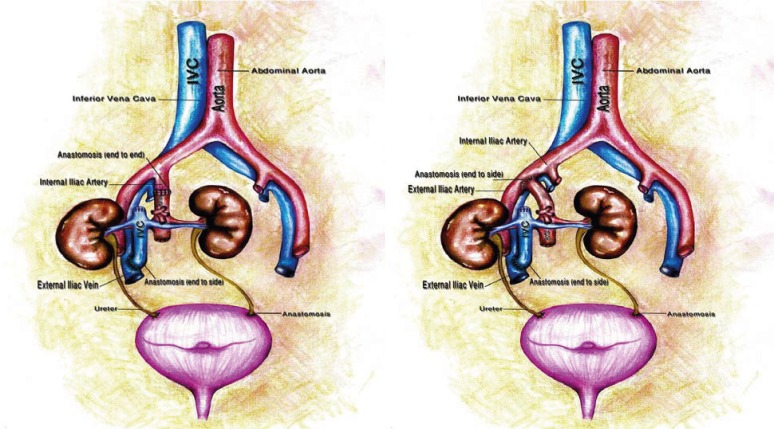
En-bloc anastomosis of two kidneys by anastomosing the aorta and the IVC to the internal iliac artery (left) or external iliac artery (right) and external iliac vein. Two ureters are implanted to urinary bladder separately

The kidneys were harvested en bloc with the aorta and the inferior vena cava (IVC). At bench surgery, the proximal end of the IVC—above the site of entrance of renal veins—was closed with running sutures (5-0 prolene); in the meantime, the distal end of the aorta—below the opening of the renal arteries—was closed. All non-renal tributaries of the aorta were ligated. In the recipient, the procedure began through a Gibson incision (hocky stick) on the right side. Retroperitoneum was entered by medial sliding of the peritoneum. Internal (or external) iliac artery and external iliac vein were mobilized. The proximal end of the aorta was anastomosed end-to-end to internal iliac artery or end-to-side to external iliac artery. Then, the distal part of the IVC was anastomosed end-to-side to external iliac vein. Vascular anastomoses were performed by continuous 6-0 prolene sutures. After revascularization, the right kidney was positioned in right iliac fossa and the left kidney was placed in space between the urinary bladder and right pelvic wall (Fig 1). Both ureters were implanted to the urinary bladder separately (by 5-0 PDS sutures) according to the Lich-Gregoire technique with a ureteral stent for each ureter. After insertion of a closed suction drain, the wound was closed anatomically. All patients received triple immunosuppression drugs including tacrolimus, mycophenolate mofetil and steroid.

Color Doppler ultrasonography was performed daily starting immediately after transplantation. Blood chemistries and serum creatinine were checked daily. Urinalysis and urine culture were requested every other day. DTPA renal scan was performed one and three months post-operation.

## RESULTS

The mean operative time was 180 (range: 165–210) minutes. All patients developed adequate urine output and their serum creatinine gradually deceased. The drains and internal foley catheters were removed seven days after surgery. The ureteral stents were removed three weeks post-operation. In the hospital course, color Doppler sonography of the transplanted kidneys revealed good arterial and venous blood flow without any thrombosis. No hydronephrosis, fluid collection or urine leakage was observed during the hospital course and follow up. DTPA renal scan showed good perfusion and function of both kidneys in all patients.

The mean serum creatinine level was 1.5 (range: 1–2.4) mg/dL at the time of discharge and 1.2 (range: 0.5–2.1) mg/dL after 2–6 months of follow up.

## DISCUSSION

The shortage of kidney donor is a rate-limiting step in renal transplantation programs in many transplant centers. To overcome the disparity between supply and demand of organs, various strategies such as the increased use of organs from ECD, have proposed. DKT is another approach to expand the existing deceased donor pool. The number of functioning nephrons is the most important determinant of kidney function; therefore, the higher number of functioning nephrons supplied by dual marginal kidneys should slow down or even prevent progressive deterioration of graft function [1].

Transplantation of two marginal kidneys rather than one suboptimal kidney to one recipient would result in more functioning nephrons that ultimately may improve the patient and graft outcome [1].

The decision to perform DKT is based on gross characteristics of the kidneys and results of renal biopsy. Small kidneys or those with extensive surface scar or cystic lesions are discarded. In renal biopsy, the important parameters for transplanting single or dual kidney are glomerulosclerosis, tubular atrophy, interstitial fibrosis and vascular narrowing [2].

The first DKT from an adult deceased donor was reported in 1996 by Johnson, *et al* [3]. The classic surgical technique for DKT included bilateral placement of the two kidneys extraperitoneally through two separate Gibson incisions or intraperitoneally through a midline incision.

Unilateral DKT was first reported by Masson, *et al*, in 1998 [4]. There are various surgical techniques for unilateral DKT [5-7]. Ekser, *et al*, proposed sequential DKT: one kidney with its vessels anastomosed end-to-side to recipient vessels (external iliac artery and vein); then another kidney with its vessels anastomosed to external iliac artery and vein distal to the site of first anastomosis [8].

Veroux, *et al*, proposed another technique for DKT. They joined the arteries and veins of the two kidneys at bench surgery. The newly joined artery and vein of the two kidneys were then anastomosed end-to-side to common iliac artery and external iliac vein, respectively [9].

Nghiem described another technique for unilateral DKT. He joined both renal arteries patch together and right renal vein implanted end-to-side to the left renal vein; then, he joined arterial patch and left renal vein anastomosed to the external iliac artery and vein, respectively [10].

In conclusion, DKT is an alternative method of expanding the donor pool. Although unilateral DKT is a complex surgical procedure, it can reduce the cold ischemia time and the operating time, leaving the contralateral side intact for further transplant. On the other hand, kidneys with multiple arteries and veins can be transplanted like a single kidney.

We described a technique of unilateral DKT that seems to be simple and fast in comparison to other DKT techniques, particularly in extremely young donor. Due to less tissue dissection in this technique than other unilateral DKT techniques, it seems that the rate of some complications such as development of lymphocele does not significantly different from that of single kidney transplantation.
